# Establishment of a small animal tumour model for *in vivo* studies with low energy laser accelerated particles

**DOI:** 10.1186/1748-717X-9-57

**Published:** 2014-02-18

**Authors:** Kerstin Brüchner, Elke Beyreuther, Michael Baumann, Mechthild Krause, Melanie Oppelt, Jörg Pawelke

**Affiliations:** 1 Department of Radiation Oncology, University Hospital Carl Gustav Carus, TU Dresden, Fetscherstr. 74, 01307 Dresden, Germany; 2 OncoRay – National Center for Radiation Research in Oncology, TU Dresden, Fetscherstr. 74, 01307 Dresden, Germany; 3 Helmholtz-Zentrum Dresden-Rossendorf, Postfach 510119, 01314 Dresden, Germany; 4 German Consortium for Translational Cancer Research (DKTK) Dresden, Fetscherstr 74, 01307 Dresden, Germany; 5 German Cancer Research Center (DKFZ) Heidelberg, Im Neuenheimer Feld 280, 69120 Heidelberg, Germany

**Keywords:** (3–10) laser particle acceleration, Small animal model, Low energy protons, Proton radiotherapy, Nude mice, KHT mouse sarcoma

## Abstract

**Background:**

The long-term aim of developing a laser based acceleration of protons and ions towards clinical application requires not only substantial technological progress, but also the radiobiological characterization of the resulting ultra-short pulsed particle beams. Recent *in vitro* data showed similar effects of laser-accelerated versus "conventional" protons on clonogenic cell survival. As the proton energies currently achieved by laser driven acceleration are too low to penetrate standard tumour models on mouse legs, the aim of the present work was to establish a tumour model allowing for the penetration of low energy protons (~ 20 MeV) to further verify their effects *in vivo*.

**Methods:**

KHT mouse sarcoma cells were injected subcutaneously in the right ear of NMRI (nu/nu) mice and the growing tumours were characterized with respect to growth parameters, histology and radiation response. In parallel, the laser system JETI was prepared for animal experimentation, i.e. a new irradiation setup was implemented and the laser parameters were carefully adjusted. Finally, a proof-of-principle experiment with laser accelerated electrons was performed to validate the tumour model under realistic conditions, i.e. altered environment and horizontal beam delivery.

**Results:**

KHT sarcoma on mice ears showed a high take rate and continuous tumour growth after reaching a volume of ~ 5 mm^3^. The first irradiation experiment using laser accelerated electrons versus 200 kV X-rays was successfully performed and tumour growth delay was evaluated. Comparable tumour growth delay was found between X-ray and laser accelerated electron irradiation. Moreover, experimental influences, like anaesthesia and positioning at JETI, were found to be negligible.

**Conclusion:**

A small animal tumour model suitable for the irradiation with low energy particles was established and validated at a laser based particle accelerator. Thus, the translation from *in vitro* to *in vivo* experimentation was for the first time realized allowing a broader preclinical validation of radiobiological characteristics and efficacy of laser driven particle accelerators in the future.

## Background

Due to their physical properties, like the inverse dose profile in tissue with an energy dependent dose deposition maximum (Bragg peak) at the end of their range, radiotherapy with protons and light ions is widely accepted to have a high potential for improving cancer treatment. However, the present accelerator technique for protons and ions demands huge buildings and high investment costs that prevent their widespread clinical application. As one potential alternative to the classical electromagnetic accelerators, particle acceleration by means of high intensity laser is under investigation [1,2]. This includes not only the technological development of high intensity lasers [3], but also extensive translational research to transfer laser based particle accelerators to clinical application. Compared to conventionally accelerated particle beams, the laser driven beams are particularly characterized by ultra-short (~ ps) particle pulses (bunches) with an ultra-high pulse dose rate (~ 10^12^ Gy min^-1^), beside broad energy spectra, low pulse frequency (repetition rate) and substantial intensity (charge) fluctuations from pulse to pulse. Along with the evaluation of dose delivery (beam transport and monitoring, dosimetry) and quality assurance, the biological effects on cell survival and tumour growth of laser driven particle beams have to be investigated for the whole translational research chain from basic cell experiments to animal studies and finally clinical trials [4].

Several groups have performed *in vitro* studies on the radiobiological effectiveness of laser accelerated electrons [5] and protons [6-10]. Summarizing these studies, no significant differences have been revealed in the radiobiological response of laser driven particle beams relative to conventional accelerated particle beams [5-10]. Similar results were obtained using a Tandem accelerator to mimic and compare the pulse dose rates of laser driven and conventional proton beams [11,12]. If these results can be validated *in vivo*, the findings would allow transferring the existing database on RBE (relative biological effectiveness) values of conventional accelerated particle beams to their laser driven equivalents.

So far no *in vivo* data have been published on laser accelerated particles. A major limitation for the *in vivo* application is the delivery of particle energies high enough to fully penetrate standard experimental tumours. Particularly, for laser accelerated protons, the available maximum energies of ~ 20 MeV are still too low for the homogeneous irradiation of a tumour transplanted into experimental animals. In consequence, the aim of the present study is the establishment of a tumour model that is small enough to be fully penetrated by ~ 20 MeV protons. For this, KHT mouse sarcoma cells were injected subcutaneously in mouse ears and the growing tumours were characterized with respect to growth parameters and histology; their radiation response was determined after 200 kV X-ray irradiation.

Finally, the applicability of the tumour model for systematic experiments at a laser accelerator was validated under the realistic and challenging conditions, like horizontal beam delivery and altered environment, at the experimental laser system JETI (Jena Titanium:Sapphire [13,14]). Although the new tumour model was motivated by future proton trials, the proof-of-principle experiment was performed with laser accelerated electrons, since the homogeneous delivery of prescribed doses to a 3D target volume is easier for electrons than for protons. Therefore, a complete irradiation setup including beam transport and control, tumour positioning and delivery of prescribed doses was implemented at JETI [15]. In parallel to the JETI laser experiments, animal irradiation with 200 kV X-rays were performed providing reference data for the laser electrons and establishing a relation to previous 200 kV X-ray data.

## Methods

### Animals and tumour model

All experiments were performed using 7-to-14-week-old female and male NMRI (nu/nu) mice continuously purchased from the Experimental Centre of the Medical Faculty of the Technische Universität Dresden. The animal facilities and the experiments at both experimental places in Dresden and Jena were approved according to the corresponding institutional guidelines and the German animal welfare regulations. The animal housing provided a 12 h light - 12 h dark cycle, a constant temperature of 26°C and a relative humidity of 50–60%. The mice were fed with commercial laboratory animal diet and water *ad libitum*. In order to further suppress their immune response, the mice were whole-body irradiated (WBI) with 4 Gy (200 kV X-rays, 20 mA, ~ 1 Gy min^-1^) two to four days before cell injection.

The KHT mouse sarcoma, originally described by Kallman et al. [16], was kindly provided by Richard Hill from the Ontario Cancer Institute in Toronto, Canada, for the present experiments. KHT cells were maintained in AlphaMEM with nucleosides supplemented with 10% FCS (foetal calf serum) and with 2 mM L-Alanyl-L-Glutamin (all from Biochrom AG, Berlin, Germany). Adapting the tumour model of Suit et al. [17], 500 KHT cells were suspended in about 50 μl PBS (phosphate buffered saline, PAA, Parching, Austria) and were injected subcutaneously between the cartilage and the skin in the middle of the right ears of anaesthetised mice. After injection the mice were continuously controlled for tumour growth firstly by visual observation and then, after reaching the starting size for the experiment, by using a calliper. For the first experiments at the 200 kV X-ray tube, the animals were included with tumour diameters of 1 – 1.5 mm. Later on, the starting size was adjusted to 2 – 2.5 mm in order to facilitate a better handling and positioning for the proof-of-principle experiment at the JETI laser accelerator.

Moreover, some of the animals were sacrificed at this time point and their tumours were extracted and immediately frozen in liquid nitrogen. The histology of the tumours was characterized by standard haematoxylin and eosin (H&E) staining. Besides, the tumour microenvironment was analysed using different immunofluorescence markers to visualize hypoxia, perfusion and vascular endothelium. The hypoxia marker pimonidazole (intraperitoneally, 0.1 mg g^-1^ body weight, dissolved at 10 mg ml^-1^ in 0.9% NaCl; Natural Pharmacia Int., Research Triangle Park, NC, USA) and the perfusion marker Hoechst 33342 (intravenously, 150 μl per animal, 6 μg ml^-1^ dissolved in PBS; Sigma Aldrich, Deisenhofen, Germany) were injected one hour and one minute before tumour excision, respectively. For staining, 10 μm thick frozen sections of the tumours were cut, air dried and fixed in acetone (4°C). Finally, antibodies against pimonidazole (Hydroxyprobe^TM^-1Mab1, clone: 4.3.11.3, 1:2500, Chemicon® Int., Temecula, Ca, USA) and against CD31 as marker for the vascular endothelium (cluster of differentiation 31, clone: MEC 13.3, 1:500, BD Pharmingen, Heidelberg, Germany) were applied.

### General experiment procedures

**Figure 1:**
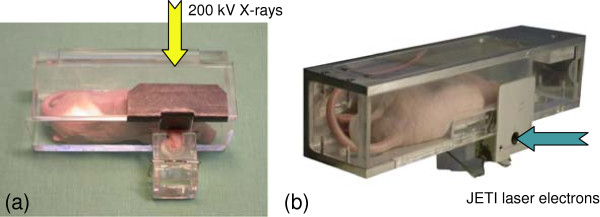
**Mouse positioning at the two radiation sources.** Mouse setup boxes for mouse fixation and reproducible tumour positioning at the 200 kV X-ray tubes **(a)** and at the JETI laser accelerator **(b)**. The mice were placed dorsoventral in the mouse setup boxes gently fixing the right ears on the PMMA block right outside of the box. **(a)** For irradiation at the vertical 200 kV X-ray beam the boxes were placed at defined positions under the collimator (not shown) allowing tumour irradiation under protection of the animal. **(b)** The horizontal electron beam delivery at JETI requires the tilt of the setup box so, that the right ear points downwards into the irradiation field. Moreover, cooling of the animals was avoided by heating up the setup boxes at JETI [15].

Prior to irradiation, the animals were anaesthetised intraperitoneally with 10 mg kg^-1^ Xylazin (Rompun®, Bayer Health Care GmbH, Leverkusen, Germany) and 100 mg kg^-1^ Ketamin (Ketamin 500 Curamed®, Curamed Pharma, Karlsruhe, Germany). The anaesthetised animals were positioned dorsoventral in specifically constructed mouse setup boxes (Figure 1), and their right ears were gently fixed at a defined position on the attached PMMA (polymethylmethacrylate) block allowing for the precise irradiation of the tumour [15]. The mouse setup boxes and their positioning at the 200 kV X-ray tube and at the JETI laser accelerator, respectively, are described below.

In parallel to the untreated controls, an additional "0 Gy" group was introduced for all irradiation experiments. Mice of this group were anaesthetised and positioned in mouse setup boxes, like those of the other dose groups, but sham irradiated. By comparing the untreated control mice, which remain in the animal laboratory, and the "0 Gy" groups, influences of the differing experimental procedures should be revealed, including anaesthesia and positioning in mouse setup boxes as well as the horizontal beam delivery and altered environment at the JETI laser accelerator.

### Animal irradiation with 200 kV X-rays

For the present experiments, a 200 kV X-ray tube of type Maxishot 200 Y.TU/320-D03 (Yxlon Int. GmbH, Hamburg, Germany) was used for WBI and for the determination of appropriate doses for the treatment of KHT sarcoma (in Dresden). The X-ray tube was operated at 20 mA and filtered inherently with 3 mm Be + 3 mm Al as well as with the additional 0.5 mm Cu external filter. Absolute dosimetry was performed with a farmer ionization chamber (Type 30010, 0.6 cm^3^ sensitive volume) that was readout by an Unidos electrometer (both PTW, Freiburg, Germany). As result, a dose rate of about 1 Gy min^-1^ was obtained for WBI of mice. Dose distributions and dose homogeneities within the irradiation fields were controlled with GafChromic EBT-1 films (ISP Corp., New York, NY, USA).

In addition to the filters, a collimator made up of 6 mm Pb and of 2 mm Al was introduced. Four collimator openings of 6 mm in diameter allow for the irradiation of ear tumours of up to four animals protecting their bodies against radiation. The anaesthetised mice were positioned in setup boxes (Figure 1a) and were placed horizontally under the collimator superimposing the fixed ears and the collimator openings. The resulting dose rate at tumour position was 0.95 Gy min^-1^. In the first trial, the animals were allocated in five different dose groups (0 Gy (4 mice), 3.8 Gy (8), 7.6 Gy (10), 9.5 Gy (8), and 13.2 Gy (7)) and one group for tumour growth control (untreated, (8)) in order to find a suitable dose range for subsequent irradiation studies.

### Experiments with laser accelerated electrons

The electron pulses were generated in a vacuum target chamber using the 30 TW laser system JETI [13,14] that delivers 28 fs short laser pulses (700 mJ energy, 800 nm central wave length) at a pulse frequency of 1 Hz. The laser pulses were focused into a hydrogen gas jet producing plasma and accelerating electrons in forward direction to a broad energy spectrum with maximum energies of a few 10 MeV. In the following, a permanent magnet system consisting of two dipole magnets (B = 0.06 T) of opposing polarity was used to filter out the radiobiological more effective low-energy electrons (< 3 MeV). After filtration, the electron beam enters the purpose-built irradiation setup that includes beam transport and control, tumour positioning and dosimetry, as described more detailed in Schürer et al. [15]. Briefly, the electron beam was collimated by a 118 mm long aluminium block with an aperture of 10 mm, prior exiting through a 1 mm thick aluminium vacuum window to propagate in air to the point of irradiation.

Based on previous experiments [5,18,19] a beam control and dosimetry system, consisting of a Bragg Peak chamber (PTW), a Faraday cup (200 mm steel, in house development and manufacturing), Gafchromic EBT-2 dosimetry films (ISP Corp., New York, NY, USA) and two electron spectrometers, was applied. For real-time beam monitoring the Bragg Peak transmission ionization chamber and the Faraday cup were deployed both providing online dose information via readout with an Unidos electrometer (PTW) and a 2.5 GHz Digital Phosphor Oscilloscope (DPO 7254, Tektronix Inc., Beaverton, OR, USA), respectively. Located in front of tumour position, EBT-2 dosimetry films were used to control the dose homogeneity over the irradiated field of about 5 mm in diameter during beam optimization and for animal irradiation. Moreover, EBT-2 films in front of each tumour allow for a retrospective determination of absolute dose administered to each individual tumour. For this, the films were calibrated for high energy electrons and readout with an Epson Perfection flatbed scanner (V750pro, Epson, Germany) [20]. Electron spectra in real-time were obtained by means of the scintillator spectrometer (in-house production, liquid scintillator BC 517H, Saint-Gobain, Valley Forge, USA), where the scintillation light was monitored in up to 40 cm water equivalent thickness (by CCD camera) providing information on the depth dose distribution and with it on the electron spectrum. Additionally, information on the low-energy part (E < 20 MeV) was ascertained by means of the mini-spectrometer. Here, the laser electrons were deflected by permanent magnets and the resulting spot positions, i.e. deflection angles, on EBT-2 films were used to reconstruct the spectrum retrospectively. To control the electron beam parameters, the two spectrometers and the Faraday cup were moved to beam position between animal irradiation. The Bragg Peak transmission chamber had a fixed position and was operated in parallel to the Faraday cup, the scintillator and to animal treatment.

For the experiments at JETI, the animals were transported from Dresden to Jena two days after injection and housed in the animal laboratory there. As a consequence of the preceding 200 kV X-ray experiments, the animals were randomly allocated in the group for tumour growth control (untreated, (7)) and in two dose groups (0 Gy (2) and 14 Gy (9)), which were applied at the JETI laser accelerator as well as at the local 200 kV X-ray tube (see below), respectively. For treatment at JETI, the anaesthetised mice were positioned in the corresponding mouse setup boxes (Figure 1b), which were tilted ahead so, that the mouse lies sideways and the tumour bearing ear points downwards allowing for tumour irradiation at the horizontal electron beam. Then, the turned box was placed in a movable docking station enabling a reproducible positioning of both, the holder and the tumour, at beam position. Positional changes of the tumour that may arise from tilting were observed by a CCD camera and corrected before irradiation. Moreover, the mouse setup boxes applied at JETI exhibited an additional lead collimator (aperture 6.5 mm) just above the tumour position to shield the animal from scattered radiation. The whole procedure at JETI, including preparation, transport, positioning and treatment, took about 20 min per mouse.

In parallel to the experiment with laser accelerated electrons 200 kV X-ray reference irradiation was performed in the local animal laboratory located in the adjacent building to the laser laboratory. As for the preceding experiments in Dresden, a 200 kV X-ray tube of type Maxishot 200 Y.TU/320-D03 (Yxlon Int. GmbH, Hamburg, Germany) was applied using the same tube parameters (20 mA, filter: 3 mm Be + 3 mm Al + 0.5 mm Cu) and an identical setup, including mouse setup boxes and additional collimator for animal irradiation, to deliver doses of 0 Gy (2 mice) and 14 Gy (3) to the mice.

After the experiment, all animals inclusive those not included in the experiment were brought back to Dresden, where the follow up took place.

### Follow up and analysis of tumour growth data

Tumour diameters were measured three times a week and the corresponding tumour volumes were calculated by the formula of an ellipsoid *π/6 x a x b*^*2*^, where *a* is the longest and *b* the shorter tumour axis perpendicular to *a*. The animals were sacrificed when the tumour reached the diameter (i.e. *a*) of about 10 mm or when the animal seemed to suffer. In order to assess the tumour growth delay between non-irradiated and irradiated animals, the time spans required to achieve the same relative volume increase after inclusion in the experiment were compared. Additionally, tumour volume doubling times (VDT) were estimated in the tumour volume range between 5 and 200 mm^3^.

For the graphical representation of tumour growth curves and the analysis of growth delay the software Microsoft Office Excel 2003 (Microsoft Corp., Redmond, WA, USA) was applied. Statistical analysis was performed using Student’s t-test and the Welch correction implemented in the software GraphPad Prism (Vers. 4, GraphPad Software Inc., La Jolla, CA, USA). Values of *p* < 0.05 were considered as statistically significant.

## Results

### Growth rates und tumour growth behaviour

**Figure 2:**
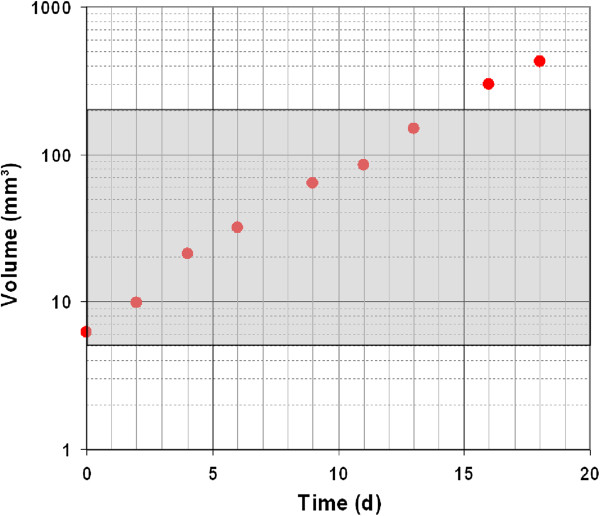
**Exponential tumour growing of KHT mouse sarcoma.** Exemplary growth curve of one KHT mouse sarcoma after injection of 500 KHT cells between the cartilage and the skin of the right mouse ear. The volume increase is shown in dependence on time starting at the tumour size at start of treatment; exponential growing is indicated for tumour volumes between 5 and 200 mm^3^ (grey coloured area).

In a first test, the number of KHT cells in the 50 μl injection volume was varied between 500 and 10000 cells to find the optimum number for a reproducible and stable growth of a single tumour on the ear. KHT cells showed a high tumour take rate, and the minimum number of 500 cells was used in the following to reduce unintended reflux of the injected cell suspension. Based on daily controls first tumour growth was observed from day 11 after injection. Besides, 45 out of 46 animals developed tumours of 1–1.5 mm in diameter, most of them between days 11 to 15 after injection. Exponential tumour growth with tumour VDT of 1.5 – 3 days was found for volumes of up to 200 mm^3^ (Figure 2).

**Figure 3:**
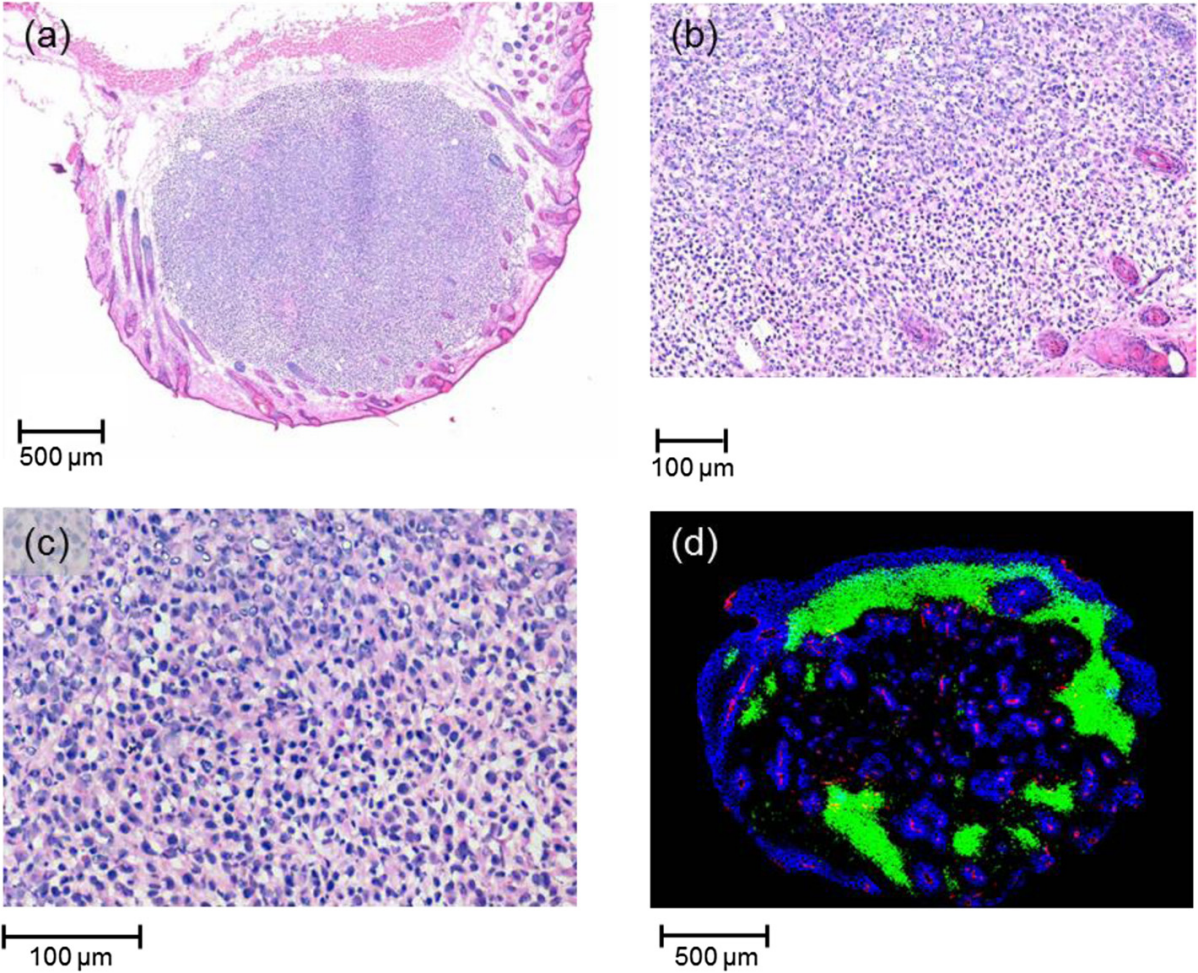
**Characterization of the tumour micromilieu at starting size.** Representative pictures of KHT mouse sarcoma characterized at start of treatment with a size of 2 mm in diameter: **(a)** The limited and bordered tumour volume was confirmed by standard H&E staining, showing **(b + c)** the integrity of cellular components by differentiating the basophil stained nuclei from the eosinophil stained cytoplasm, whereas necrosis of single cells cannot be excluded (Figures **b** and **c** are higher magnifications of Figure **a**). **(d)** The analysis of the tumour histology revealed a well perfused tumour (blue, Hoechst 33342) that is interspersed with small vessels (red, CD31) and some hypoxic area (green, pimonidazole).

By means of standard H&E staining the tumour histology, i.e. mouse sarcoma, was confirmed. The tumours were found to be solid and circumscribed (Figure 3a), growing subcutaneously without infiltration of the skin. Necrotic areas were not detected (Figures 3b and c). Using immunofluorescence analysis (Figure 3d) evenly distributed perfused areas, but also larger hypoxic regions were revealed. Tumour vessels were observed mainly in perfused, but also in unperfused, hypoxic areas.

### Dose range specification

**Figure 4:**
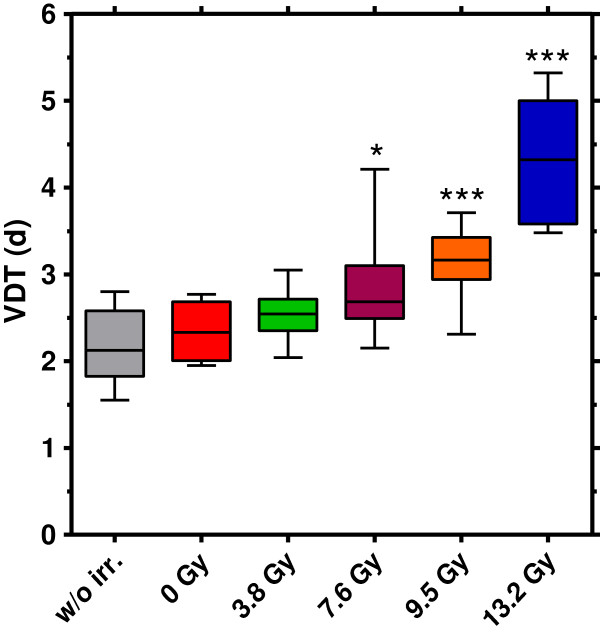
**Dose dependent tumour volume doubling times for 200 kV X-ray irradiation.** KHT tumour volume doubling times for the unirradiated control (8 mice) and after irradiation with 200 kV X-rays (0 Gy (4); 3.8 Gy (8); 7.6 Gy (10); 9.5 Gy (8); 13.2 Gy (7)) determined on basis of the tumour volume at which the animals were included in the experiment. Compared to the unirradiated control the VDT values for doses of 7.5 Gy and higher were significantly increased (*p < 0.05, ***p < 0.001).

**Figure 5:**
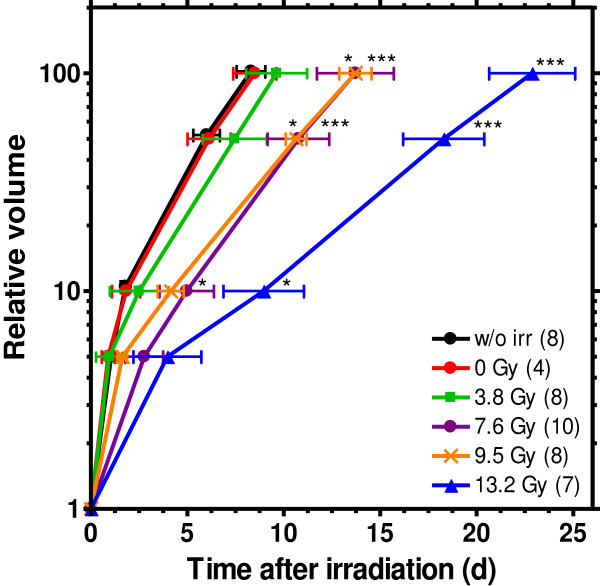
**Dose dependent growth delay induced by 200 kV X-ray treatment.** Tumour growth curves measured after irradiation with 200 kV X-rays (0 – 13.2 Gy) and for the untreated control (black curve, partly covered by the 0 Gy line). The relative volumes in dependence on time are determined on basis of the volumes at the start of the treatment. The numbers of animals allocated to the different groups are given in brackets; the measured data are given as mean value ± SEM of all animals in this particular group. The tumour growth delay relative to the untreated control was significant (*p < 0.05, ***p < 0.001) for doses higher than 7.6 Gy for the 10fold volume increase and for doses higher than 3.8 Gy at higher volume increases, respectively.

Sufficient studies on tumour growth delay require the definition of reasonable radiation doses leading to a significant growth delay compared to untreated tumours, but no permanent tumour control. The VDT obtained for the KHT tumours after 200 kV X-ray treatment in Dresden are summarized in Figure 4, displaying a dose-dependent retardation of tumour growth. Correspondingly, Figure 5 shows a clear tumour growth delay, measured as the time necessary to reach for example 10fold the volume at time of treatment in comparison to the untreated controls. For doses higher than 7.6 Gy this increase was significant with *p*-values of p = 0.013 at a dose of 9.5 Gy and p = 0.015 at the highest dose of 13.2 Gy, respectively. No radiation side effects and no tumour control were noticed for the whole dose range. No differences in VDT or tumour growth delay were detected between the untreated control group and the sham irradiated group. Consequently, the handling procedure at the 200 kV X-ray tube, i.e. anaesthesia, positioning and persistence in the mouse setup boxes, has no additional impact on tumour growth.

### Proof-of-principle experiment at JETI

After the results of the 200 kV X-ray study a dose of 13.2 Gy should also be applied at the experiments in Jena. However, for technical reasons, the highest dose was adjusted to 14 Gy for both the irradiation with laser accelerated electrons and with 200 kV X-rays, respectively. The sham irradiated "0 Gy" dose groups were retained, since the laboratory conditions and the animal positioning were clearly different at the two radiation sources.

At JETI the prescribed dose of 14 Gy was delivered with a mean dose rate of 1 Gy min^-1^ by few hundreds of laser accelerated electron pulses. The retrospective determination of absolute dose administered to an individual tumour by GafChromic EBT-2 film comes along with an overall dose uncertainty of 8%, whereas a dose uncertainty of 5% was linked with a single film dose measurement. Furthermore, a maximum dose inhomogeneity over the irradiated field of 6% was measured by EBT-2 dosimetry film located in front of the tumour position for all irradiations. The depth dose distribution was controlled by film stack irradiation before and after mouse irradiation, showing a maximum inhomogeneity of 3%. One prerequisite for the delivery of the prescribed dose was the online dose monitoring via Bragg Peak transmission chamber and the Faraday cup. By beam tuning and dose calibration (ionization chamber and Faraday cup readout to absolute dose) on a daily basis before starting mouse irradiation a maximum variation of the actual delivered dose of 5% was achieved.

**Figure 6:**
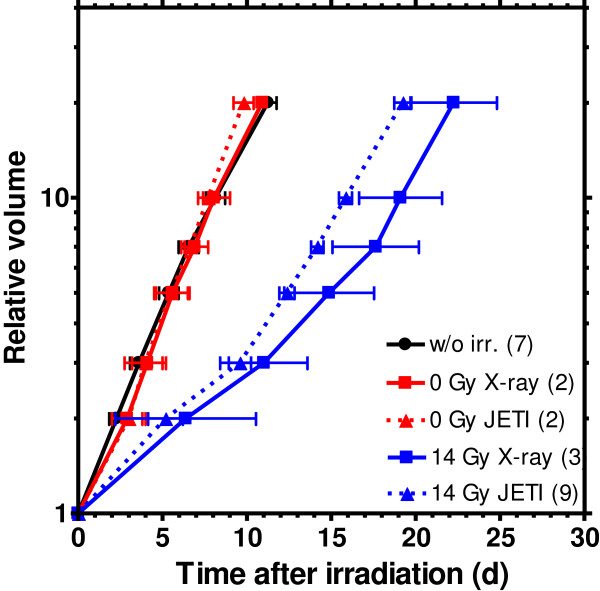
**Comparison of tumour growth after irradiation with laser electrons and 200 kV X-rays.** Time dependent relative tumour volume increase for the untreated control, sham irradiation with "0 Gy" and after treatment with a dose of 14 Gy by JETI laser accelerated electrons (triangles) and by 200 kV X-rays (squares), respectively. The numbers in brackets specify the animals allocated to the different groups, the error bars display the corresponding SEM to the mean value of the corresponding group. The apparently different tumour growth rates, compared to the results displayed in Figure 5, resulted from the different tumour diameters of 1.0 – 1.5 mm and 2.0 – 2.5 mm defined as starting size at which the animals were included in the experiments in Dresden and Jena, respectively.

Comparing the growth curves of the unirradiated control and the sham-irradiated "0 Gy" dose groups obtained for the two radiation sources, no apparent difference was observed (Figure 6). In consequence, the whole handling procedure at JETI including the horizontal electron beam delivery that come along with the tilt of animal and tumour as well as the altered conditions in a physical laboratory, has no impact on tumour growth. The comparison of the 14 Gy growth curves reveals no significant difference (p > 0.3) between the times needed to reach the 5fold to 100fold volume increase after irradiation with laser accelerated electrons and 200 kV X-rays, respectively (Figure 6).

## Discussion

The integration of laser based proton accelerators in radiotherapy clinics is a long-term aim that includes not only the technological development of high intensity lasers and new techniques for dose delivery, but also comprehensive research on the radiobiological consequences of the new beam quality, i.e. ultra-short, ultra-intense particle pulses. Considering the translational research chain [4] forgoing *in vitro* experiments with laser accelerated protons [6-10] have to be followed by animal studies, which are however limited by the still low proton energies at currently available laser driven accelerators.

The present paper describes the establishment of a small animal tumour model that allows for the penetration by ~ 20 MeV protons. For this, an anterior mouse ear tumour model [17] was successfully adopted. The choice of the fast-growing KHT mouse sarcoma model for injection was likely responsible for the very high tumour growth rate of more than 95%. Besides this remarkable high growth rate, the tumour model exhibited continuous growth behaviour with a distinguished exponential phase (Figure 2) that can be applied for tumour growth rate analyses. Moreover, a significant dose dependent tumour growth delay was found after 200 kV X-ray treatment, fulfilling a basic requirement for the performance of irradiation studies. Also important for this purpose was the absence of radiation side effects and of tumour control, even at the highest dose of 13.2 Gy, as well as of impacts by the experimental procedures like anaesthesia and positioning in the mouse holder during irradiation. Beside these outcomes, an important technological prerequisite was the ability of the laser system JETI to deliver such a high dose in a reasonable time. Indeed, with the newly implemented setup for small animal irradiation [15] and by careful tuning of the laser parameters the irradiation times could be limited to a few minutes minimizing the overall treatment time and therefore reducing anaesthesia.

Considering the established tumour model in comparison to the standard subcutaneous tumour model on mouse legs, one obvious difference is the necessity of a small tumour volume at the time of treatment (~ 5 mm^3^) and the corresponding tumour diameter of approx. 2 mm that allows for the penetration by ~ 20 MeV protons. By contrast, considerably higher tumour diameters of about 7 mm and tumour volumes of ~ 180 mm^3^ are routinely used for the standard model [21-24]. One question arising in this context deals with the constitution of the tumour and its microenvironment. Investigating tumours at the size of 5 mm^3^, immunofluorescence analysis revealed that already the small tumours used in the present experiments interact with the surrounding tissue and activate endothelial cells to form vessels. And, similar to larger tumours, the KHT sarcomas also generate hypoxia, which might explain their relatively high radioresistance with a tumour control dose higher than 14 Gy.

Finally, after fulfilling all the biological and technological requirements [14,15], a proof-of-principle experiment at a laser accelerator was performed using the laser facility JETI in Jena. Since the main goal of this study was the validation of the tumour model under realistic conditions at a laser accelerator, only two dose values were applied (sham "0 Gy" and 14 Gy). One important issue for the evaluation of the suitability of the model at an experimental laser accelerator was the impact of the experimental conditions that clearly differ from those at the reference 200 kV X-ray tube. Comparing the growth curves obtained after "0 Gy irradiation" at both radiation sources and for the untreated control, no difference could be observed between all of them. Although the statistic of this first experiment was quite low, these data indicate that the impact of anaesthesia and positioning in mouse setup boxes, of the altered environment, i.e. temperature and moisture differing from animal laboratory, and of the horizontal irradiation at JETI on the tumour response can be neglected. Nevertheless, this finding awaits confirmation in ongoing full scale experiments [14].

Further full scale experiments with different radiation dose groups are also necessary to allow drawing conclusions on the RBE of laser accelerated electrons. For the present proof-of-principle study the comparison of the measured growth delays reveals by trend a higher radiobiological effectiveness for 200 kV X-rays relative to laser accelerated electrons, which may be explained by known higher RBE of kV X-ray irradiation compared to MV photons [25,26] or electrons [26].

In a next step, the new mouse ear tumour model should be validated for experimentation with laser accelerated protons in order to confirm the preliminary results obtained with laser accelerated electrons and to prepare future full scale studies with laser accelerated protons. Moreover, the new model should also be evaluated for human tumours and the choice of radiobiological endpoint should be reconsidered. For the first trial, tumour growth delay was a simple but nonetheless meaningful parameter, whereas tumour control studies as more valid endpoint in clinical cancer research [27,28] should be considered for the next translational steps.

## Conclusions

A small animal tumour model suitable for the irradiation with low energy particles was established and validated for systematic experimentation at a laser based electron accelerator. Thereby, the translation from *in vitro* to *in vivo* studies was for the first time realized and the experiences and the implemented setup provide a valuable basis for the experimentation with laser accelerated protons.

## Abbreviations

CCD: Charged coupled device; CD31: Cluster of differentiation 31; FCS: Foetal calf serum; H&E: Haematoxylin and eosin; JETI: Jena Titanium:Sapphire laser system; PBS: Phosphate buffered saline; PMMA: Polymethylmethacrylate; RBE: Relative biological effectiveness; SEM: Standard error of the mean; TW: Terawatt; VDT: Volume doubling time(s); WBI: Whole body irradiation.

## Competing interests

The authors declare that they have no competing interests.

## Authors’ contributions

KB – participated in the design and performed all of the animal studies, performed data analysis and helped to draft the manuscript; EB – drafted the manuscript and participated in the animal studies; MB – contributed with regard to study design and scientific context; MK – contributed with regard to study design and scientific context; MO – participated in the animal studies and data analysis, JP – contributed with regard to study design and scientific context, helped to draft the manuscript; All authors read and approved the final manuscript.
